# Establishment and characterization of a multi-drug resistant cell line for canine mammary tumors

**DOI:** 10.3389/fvets.2023.1129756

**Published:** 2023-03-30

**Authors:** Chaoyu Zhou, Zixiang Lin, Xinqiu Li, Di Zhang, Peijia Song

**Affiliations:** Department of Veterinary Clinical Science, College of Veterinary Medicine, China Agricultural University, Beijing, China

**Keywords:** canine mammary tumor (CMT), chemotherapy, multidrug resistance (MDR), Breast Cancer Resistance Protein (BCRP or ABCG2), mRNA-sequencing (mRNA-seq)

## Abstract

**Background and purpose:**

Canine mammary tumors are the most common tumor disease of female dogs, and adjuvant chemotherapy often results in multi-drug resistance. Currently, the mechanisms underlying the development of tumor multi-drug resistance are unclear. The translation of research applications that can be used to effectively overcome tumor resistance is similarly hampered. Therefore, it is urgent to construct multi-drug resistance models of canine mammary tumors that can be used for research, to explore the mechanisms and means of overcoming resistance.

**Materials and methods:**

In this study, the canine triple negative breast cancer cell line CMT-7364 was induced to develop multidrug resistance using doxorubicin by high-dose drug pulse method. The drug resistance and the expression of drug transport pumps of the cells was verified by CCK8 assay, immunoblotting, qPCR and immunofluorescence. Next, we used scratch assay and Transwell invasion assay to compare the migration and invasion abilities of the two cell lines and examined the expression of EMT-related proteins in both using immunoblotting. The differences of transcriptome between parental and drug-resistant cell lines were detected by RNA-seq sequencing. Finally, mouse xenograft models of drug-resistant and parental cell lines were constructed to evaluate the tumorigenic ability.

**Results:**

After more than 50 generations of continuous passages stimulated by high-dose drug pulse method, the morphology of drug-resistant cell line CMT-7364/R tended to be mesenchymal-like and heterogeneous under light microscopy compared with the parental cell line CMT-7364/S, and developed resistance to doxorubicin and other commonly used chemotherapeutic drugs. In CMT-7364/R, BCRP was expressed at higher levels at both transcriptional and protein levels, while P-glycoprotein was not significantly different. Secondly, the migration and invasion ability of CMT-7364/R was significantly enhanced, with decreased expression of E-cadherin and increased expression of vimentin and mucin 1-N terminus. Finally, mouse xenograft models were constructed, while there was no significant difference in the volume of masses formed at 21 days.

**Conclusion:**

In summary, by using the canine mammary tumor cell line CMT-7364/S as the parental cell line, we successfully constructed a multidrug-resistant CMT-7364/R with high-dose drug pulse methods. Compared to its parental cell line, CMT-7364/R has decreased growth rate, overexpression of BCRP and increased migration and invasion ability due to EMT. The results of this study showed that CMT-7364/R might serve as a model for future studies on tumor drug resistance.

## 1. Introduction

Canine mammary tumors (CMTs) are the most common tumors in female dogs, accounting for 50–70% of all tumors. Of these, canine mammary cancer (CMC) accounts for 50% of CMTs (1–3). CMTs are highly heterogeneous group of tumors with a prevalence that varies by geographic region and is higher in countries where ovariectomy is not widely available (4). Surgery is currently the main treatment for the control of CMTs, with the aim of removing the tumor and ensuring that the margins are intact and free of residual tumor cells and, as appropriate, preventing new tumors from developing in the remaining gland. After tumor resection surgery, usually, combined with adjuvant chemotherapy can reduce the risk of tumor recurrence and metastasis.

Although the utility of chemotherapy has not been confirmed by large-scale studies, chemotherapy remains the best adjuvant option after surgical resection due to the highly heterogeneous and aggressive nature of CMTs. Unfortunately, however, tumor multi-drug resistance often causes chemotherapy failure (5). Therefore, in recent years, great efforts have been made to elucidate the mechanisms of tumor drug resistance in order to find new molecular targets. It is clear that the development of drug resistance in CMTs is multifaceted and based on complex interactions between the tumor microenvironment, drug efflux, tumor stem cells and solid tumor cells, among others, and is governed by alterations in multiple signaling pathways (6). One of the most important mechanisms is drug efflux mediated by the drug efflux pump, and the ATP-binding Cassette transporters (ABC transporters) uses ATP to efflux a variety of compounds through the cell membrane, including a wide range of anticancer drugs with different structures and properties (7). Many ABC transporter proteins are closely associated with chemoresistance in many solid tumors, including breast cancer, in particular, P-glycoprotein (P-gp) and Breast Cancer Resistance Protein (BCRP) (8).

The construction of chemotherapeutic drug-resistant cell lines by *in vitro* development has a long history and can be used to study the mechanisms of chemoresistance to chemotherapeutic agents. The primary method of constructing tumor-resistant cell lines in the laboratory is to repeatedly expose tumor cells to drugs during cell culture. Cells that survive this “survival stress” are screened for greater drug tolerance than their parental cells. The induction of drug resistance in cell lines *in vitro* often takes 3–18 months, and many factors are involved, including the choice of parental cell line, the type of drug used, the dose of the drug, and the method and interval of administration (9). In general, drug resistance models can be divided into two types according to the degree of resistance: clinical relevant drug-resistant cell lines and high-level laboratory models. The induction of clinically relevant drug-resistant models is designed to mimic the chemotherapy received by the patient in a clinical situation, using low doses of drugs and pulsed therapy, where cells are given intermittent “repair time” in drug-free medium, resulting in cell models with 2–8 fold resistance compared to the parental cell lines. In contrast, the induction of laboratory models with high levels of drug resistance is often done in a stepwise manner, using higher doses of drug and increasing the dose over time, with cells cultured costantly in drug-containing medium. Compared to the former, drug-resistant cell lines constructed by the stepwise method have a higher resistance multiplicity and more changes at the molecular level, allowing researchers to investigate and validate cytotoxicity and resistance mechanisms. The disadvantage is that the clinical relevance is weak (10).

Currently, the mechanisms by which tumor resistance occurs in CMTs are not clear, so it is of objective value to establish a clinically relevant and stable *in vitro* research model as a basis for exploring potential ways and means of coping with tumor drug resistance. Several methods have been used in the field of human medicine to construct *in vitro* tumor cell resistance models for multiple cancer species, but there is a paucity of such models in the field of veterinary medicine (11). Therefore, the purpose of this study is to construct a clinically relevant *in vitro* canine mammary tumor multi-drug resistance model by using doxorubicin as a stimulus and high-dose drug pulse method. Hoping to provide a research basis for investigating the mechanism of drug resistance and overcoming drug resistance in CMTs.

## 2. Materials and methods

### 2.1. Cell line and cell culture

The CMT-7364 (Veterinary Teaching Hospital, China Agricultural University, Beijing, China) canine mammary tumor cell lines is derived from a clinically spontaneous canine breast tumor. The case was a 13-year-old female dog with a histopathological diagnosis of grade III ductal papillary and triple negative mammary carcinoma. After primary isolation and culture, a stable passaged cell line has been formed and cultured in DMEM medium containing 10% FBS (Fetal Bovine Serum) and 1% penicillin-streptomycin in a culture environment with a temperature of 37°C and a CO_2_ content of 5% in our former study (12). were grown in DMEM (C11995500BT, Gibco, USA) medium with 10% fetal bovine serum (FBS)(16,000,044, Gibco, USA) and penicillin (100 units/mL) and streptomycin (0.1 mg/mL)(C0222, Beyotime, China). All cells were incubated at 37°C in a humidified atmosphere with 5% CO_2_.

### 2.2. High-dose drug pulse treatment on cells

We selected CMT-7364 as the parental cell line and set up a normal passaged group and a high-dose drug pulse group. When developing the drug-resistant cell line, a high-dose drug pulse method is used. After reaching the logarithmic growth stage, cells were subjected to a drug pulse by adding doxorubicin to the medium at concentrations up to the IC_50_ value. The drug was allowed to act for a period of 48 h. In a culture environment with a temperature of 37°C and a CO_2_ content of 5%, the drug-containing culture solution was discarded, then washed twice using PBS and add normal culture medium. Thereafter, liquid is changed every 1–2 days to remove metabolic waste and floating dead cells. After the surviving cells regain their ability to grow, they are passed on and the drug pulse is given again according to the above method. This cycle is repeated for at least 50 generations. We named the final cell line obtained in the normal passaged group as CMT-7364/S and the cell line in the high-dose drug pulse group as CMT-7364/R.

### 2.3. Cell growth and viability assay

CMT-7364/S and CMT-7364/R cell lines were seeded in triplicate in 96-well plates with 5 × 10^3^ and 1 × 10^4^ cells per well for cell growth Assay and cell viability assay respectively. Then the cells were incubated for 24 h to allow attachment. For cell growth assay, cells in triplicate were incubated with 10 μL volume Cell Counting Kit-8(CCK8, Solarbio Science & Technology Co., Ltd. Beijing) for 2 h and then detected in microplate reader (ELx808™; BioTek Instruments, Inc., Winooski, VT, USA) with a 450 nm filter at every 24-h interval for 7 days. When cell growth curve was plotted, the cell growth doubling time was calculated with Graphpad Prism 9. For cell viability assay, then different concentration of drugs were mixed with cells. After 48 h, a 10 μL volume of CCK8 was added to each well according to the manufacturer's insctructions, measuring the optical density (OD) in the same at 450 nm, and the IC_50_ was calculated using Graphpad Prism 9. Subsequently the resistance index was calculated according to the following equation:


(1) $$\begin{array}{l} \;Resistance\;Index\\ =IC_{50}\;of\;Resistant\;Cell\;Line/IC_{50}\;of\;Parental\;Cell\;Line \end{array}$$

### 2.4. Cell migration and transwell invasion assay

Two types of cells were seeded into a 6-well plate, each with 5 × 10^5^ cells per well and three parallel wells marked with a straight line at the bottom of each well. After the cells formed a monolayer and fully adhered to the bottom of the well, the supernatant was discarded, and a sterile 200 μL pipette tip was used to scratch the adherent cells along the marked line in a single direction, creating a linear region of detached cells, followed by washing the detached cells with sterile PBS. In this way, a regular and uniformly wide blank area was created in each well, and then 2 mL of serum-free DMEM medium was added. At this point, the width of the scratch was photographed as 0 h, and then photographs were taken at 24 h and 48 h in the same area. The images were processed using Image J software to calculate the scratch area at different time points for each group. For Transwell invasion assay, we used 8.0 μm Transwell Permeable Supports (Corning). The upper chamber was pre-coated with Matrigel Matrix (BD Biosciences), and 600 μL medium containing 10% FBS was added to the lower chamber. The cell was plated at a density of 5 × 10^4^ using 200 μL serum-free DMEM in the upper chamber. After incubation for 24 h, cells that did not invaded through the membrane were mechanically removed with a cotton swab. Next, 4% paraformaldehyde was used to fix the cells on the bottom surface of the membrane for 10 min, and then cells were stained with a 0.4% crystal violet solution. The invaded cells were imaged and quantified under microscopy.

### 2.5. Cell cycle analysis

Flow cytometry was used to determine the cell cycle distribution of chemotherapeutic-treated cells. CMT-7364/S and CMT-7364/R cell lines at logarithmic growth stage were inoculated in 12-well plates at 2.5 × 10^5^ cells/well and incubated at constant temperature for 24h to allow adequate wall attachment.The cells were treated with 5 and 10 μM doxorubicin for 24 h, respectively. After that, the cells were extracted and preserved in 70% ethanol at 4°C for 2 h. The cells were then centrifuged at 1,300 rpm for 5 min to remove the ethanol before being rinsed with PBS and centrifuged again. Using a BD FACSCalibur flow cytometer with selective gating to remove doublets of cells, the cells were stained with propidium iodide (PI) to form PI-DNA complexes in order to assess cell cycle distribution. Data were analyzed using FlowJo V10 software.

### 2.6. Western blot

Both type of cells, CMT-7364/S and CMT-7364/R were plated in a 6-well plate at 1 × 10^6^ cells per well. After fully attachment, protein extraction was performed with ice-cold lysis buffer (P0013B, Beyotime, China) following the manufacturer instructions, and then proteins were quantified using the BCA protein assay kit (P0012S, Beyotime, China). Equal protein amount (20 μg protein per lane) were seperated on a 10% SDS-PAGE gel and transferred onto polyvinylidene fluoride (PVDF) membranes (IPVH000 10, Merck Millipore). The membranes were blocked with 5% skim milk diluted with TBST for 1 h at room temperature, followed by incubation with primary antibody overnight. Primary bodies included anti-P-gp (1:1,000; Cell Signaling Technology, 13342), anti-GAPDH (1:2,000; Cell Signaling Technology,5174), anti-E-Cadherin (1:1,000; Cell Signaling Technology, 3195S), anti-Vimentin (1:1,000; Cell Signaling Technology, 5741S), anti-MUC1-N (1:2,000; Proteintech, 19976-1-AP), anti-BCRP (1:2,000; Proteintech, 27286-1-AP). Subsequently, membranes were incubated in horseradish peroxidase (HRP)-conjugated secondary antibodies, anti-rabbit IgG (1:5,000; Cell Signaling, 7074) for 1 h at 37°C Finally, using a chemiluminescence imaging analysis system (Tanon 5200, China) to detect signals. The quantification of the bands was done by Image J software.

### 2.7. Real-time PCR

Total RNA was extracted using TRIzol Up (TransGen Biotech) following the manufacturer's protocol. RNA concentrations were measured using a NanoDrop (NanoDrop Technologies). RNA was reverse transcribed to cDNA using PrimeScript™ RT reagent Kit with gDNA Eraser (Takara). The gene GAPDH was used as an internal control. The primers used for *mdr1, abcg2* and *gapdh* were provided in Additional file. All the real-time PCR reactions were performed using SYBR Premix Ex II (Tli RNaseH Plus) in Applied Biosystems 7500 Fast Real-Time PCR System (Applied Biosystems). The Ct for quantification and fold change for target genes was normalized by internal control.

### 2.8. Detection of cellular uptake of doxorubicin

Attach round glass coverslips pretreated with TC (YA0351, Solarbio) to the bottom of the wells of a 12-well plate. Both type of cells were seeded on the glass coverslips at a density of 1 × 10^5^ per well and incubated at 37°C in a humidified atmosphere containing 5% CO_2_. After the cells were fully adhered to the coverslips, the medium was discarded and 1 mL of 5 μM DOX was added to each well and incubated for 12 h, then washed the cells with PBS three times. The cells were fixed with 4% paraformaldehyde for 20 min followed by another three washes with PBS at room temperature. After that, 10 μL of antifade mounting medium with DAPI (P0131, Beyotime) was added dropwise to each coverslips for sealing. The coverslips were examined by confocal microscopy as soon as possible.

### 2.9. Immunofluorescence

Cells (1 × 10^5^ cells per well) were plated on coverslips. After 24 h, the cells were fixed in 4% paraformaldehyde solution for 20 min at room temperature, and permeabilized in 0.5% Triton X-100 for 5 min. Then, the cells were washed three times, incubated with anti-P-gp or anti-BCRP(1:500) overnight at 4°C, then incubated with appropriate conjugated secondary antibodies for 1 h at 37°C. After that, 10 μL of antifade mounting medium with DAPI(P0131, Beyotime) was added dropwise to each coverslips for sealing. The coverslips were examined by confocal microscopy as soon as possible.

### 2.10. RNA extraction and RNA sequencing analysis

Cells were seeded in triplicate into 6-well plates (1 × 10^6^ cells per well). After 24 h of incubation, cells were collected by trypsinisation, snap-frozen on dry ice and stored at –70°C for RNA extraction. The TransZol Up(ET111-01, TransGen Biotech) was used to extract RNA from the cells. Total RNA was purified from animal cells using EasyPure RNA Kit (ER101-01, TransGen Biotech), according to the manufacturer's protocol. RNA-seq data analyses were performed by DIATRE Biotechnology, Shanghai, China. All datasets generated and analyzed within this study are available in the NCBI's Sequence Read Archive by accession number PRJNA914497.

### 2.11. Tumorigenicity assay

Tumor xenografts were established in 5-week-old BALB/c nude mice (Vital River, China) by subcutaneous injection of cells into the mammary fat pad. There were three mice in each group. For each tumor, 5 × 10^6^ cells were resuspended in 200 μL PBS. The mice were monitored weekly for the growth of tumors. Tumor growth (tumor length and width) and body weights were measured every 3 days until the study was terminated on day 21. Tumor volume was calculated using formula: length x width^2^/2. At the end of the experiment, all the mice were first anesthetized with isofluorane and then euthanized via CO_2_ asphyxiation to collect xenograft tumors and major organs. All animal procedures were approved by the Institutional Animal Care and Use Committee of China Agricultural University.

### 2.12. Statistical analysis

Statistical analysis was performed and graphs were generated with GraphPad Prism 9 or SPSS. Statistical differences in this study were explored by Student's *t*-test. Data are shown as mean ± SEM. The *p* < 0.05 were deemed statistically significant.

## 3. Result

### 3.1. Successful construction of multi-drug resistant canine mammary tumor cell lines by high-dose drug shock method

**Figure 1:**
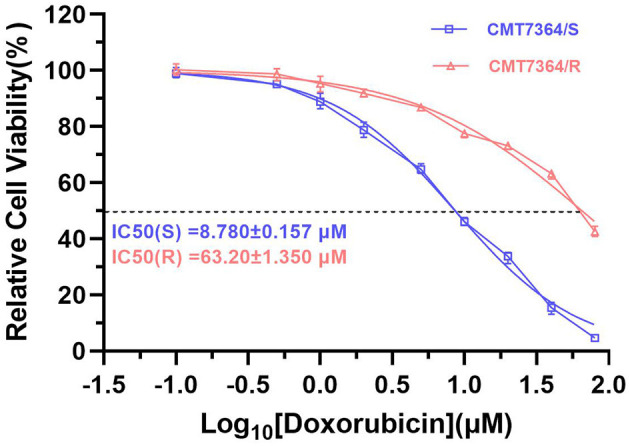
Sensitivity of drug-resistant cell lines and parental cell line to doxorubicin. Relative cell viability was determined by CCK8 assay. The cells were treated with doxorubicin for 48h. Cells treated with vehicle serve as a blank control. All experiments were conducted in triplicate and data were expressed as the mean ± SD (*n* = 3).

**Figure 2:**
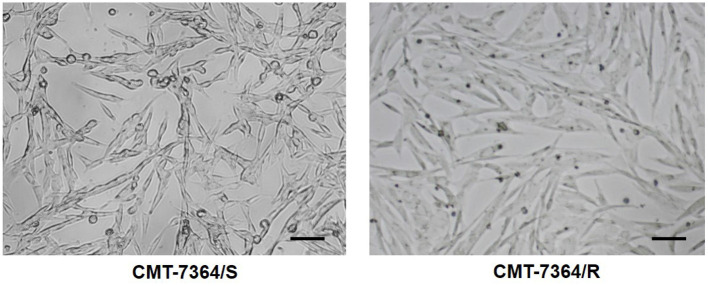
Comparison of cell morphology of drug-resistant cell lines and their parent cell lines under optical microscopy. Notice that after continuous high-dose drug pulse, the cell boundaries of CMT-7364/R are not clear and the refractivity is weak. The scale bar in the figure is 10 μm.

**Figure 3:**
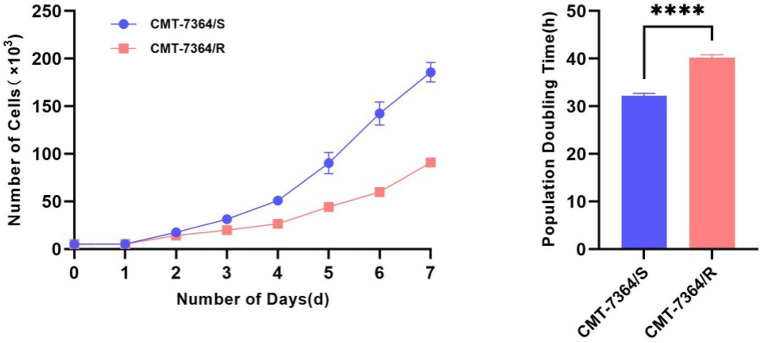
Growth curves and population doubling times of drug-resistant cell lines and their parent cell lines.The growth curve was measured at 24-h intervals for 7 consecutive days. Population doubling time (PDT) of CMT-7364/R was 40.17 ± 0.3700 h, while the PDT of its parental cell line was 32.23 ± 0.2773 h. Data were presented as the mean ± SD of three independent experiments. ****p < 0.0001.

After 50 consecutive generations of passages according to the method described in Section 2.2, we examined the sensitivity of the cells to DOX by calculating the relative survival rate of the cells. The drug-resistant cell line was less sensitive to DOX with an IC_50_ of 63.20 ± 1.350 μM compared to its parental cell line with an IC_50_ of 8.780 ± 0.157 μM and a resistance index greater than 5 (RI = 7.198), which meets the requirements for a drug-resistant cell line (Figure 1) (9). In this study, we named the *in vitro* developed drug-resistant canine mammary tumor cell line as CMT-7364/R (Resistant) and the corresponding parental cell line as CMT-7364/S (Sensitive). After more than 50 consecutive generations of high-dose drug shock induction, obvious changes have appeared in cell morphology. CMT-7364/S is mostly shuttle-shaped, with clear boundaries, uniform size, and strong refractivity, while CMT-7364/R is of heteromorphism with weak refractivity (Figure 2). During the *in vitro* cell culture process, we noticed that the growth rate of CMT-7364/R was slower than its parental cell line. To clarify the difference in proliferation ability between the two cell lines, we measured and plotted the growth curves of both, and the results are shown in Figure 3. According to this growth curve, we calculated the population doubling time (PDT) of both cell lines. Among them, the PDT of CMT-7364/R was 40.17 ± 0.3700 h, while the PDT of its parental cell line was 32.23 ± 0.2773 h (****p < 0.0001). It indicates that the proliferative capacity of CMT-7364/R was diminished compared to CMT-7364/S.

### 3.2. Validation of drug resistance

**Figure 4:**
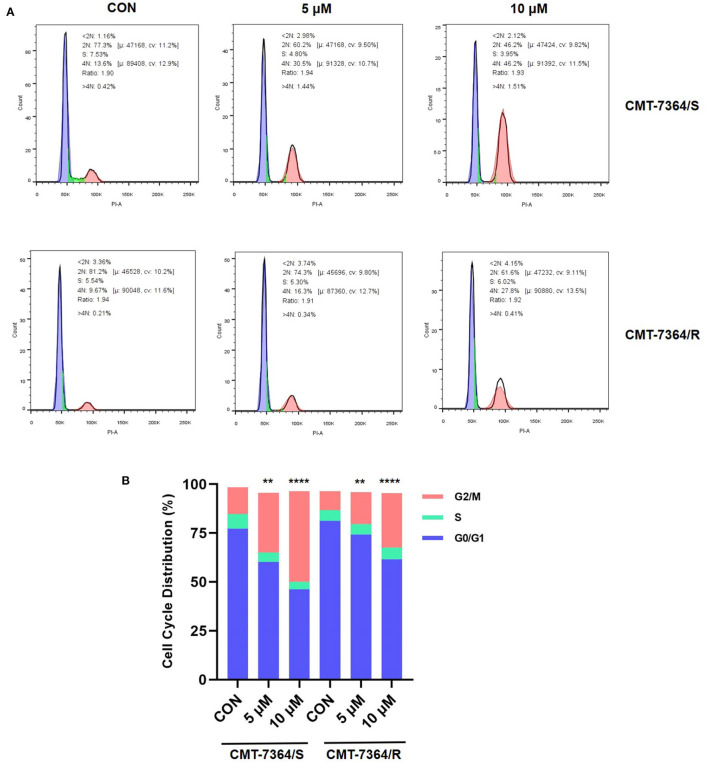
Distribution of cell cycles of drug-resistant cell lines and their parent cell lines under the action of doxorubicin at different concentrations. **(A)** Cell cycle distribution was analyzed by flow cytometry after 24 h treatment with 5 or 10 μM doxorubicin. **(B)** Statistical analysis of the cell cycle distribution showed that CMT-7364/R is more resistant to doxorubicin-induced cell cycle block. Data were presented as the mean ± SD of three independent experiments. ^**^p < 0.01, ^****^p < 0.0001.

Doxorubicin, a cycle non-specific anticancer chemotherapeutic agent, acts on cells of all phases and can cause G2/M phase cell cycle arrest. Therefore, to fully verify the resistance of CMT-7364/R to DOX, we examined the cell cycle distribution of both cell lines and the effect of DOX on them. The percentage of cells in each phase in different groups and their distribution are shown in Figure 4. Both in CMT-7364/R and CMT-7364/S, the percentage of cells in G2/M phase increased after 24h treatment with certain concentration of DOX, and the percentage of cells in G0/G1 phase was down-regulated in a dose-dependent manner. By statistical analysis, we found that the differences in the percentage of cells distributed in G2/M phase between CMT-7364/R and CMT-7364/S groups, whether treated with 5 μM or 10μM DOX, were statistically significant (*p* < 0.01 and 0.0001, respectively). This indicates that CMT-7364/R is more resistant to the cell cycle blocking effect of DOX.

**Figure 5:**
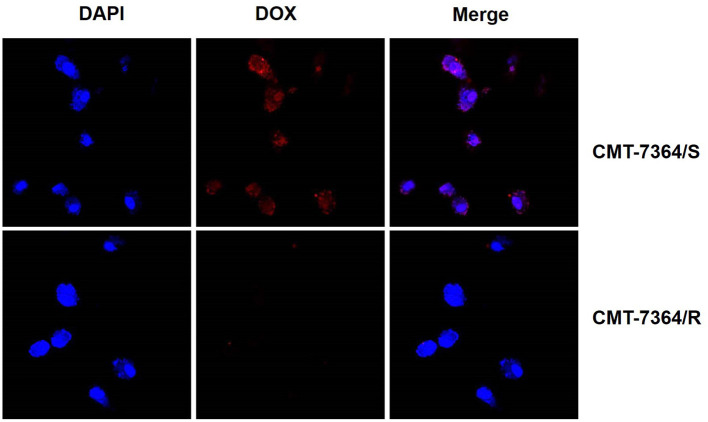
Uptake of doxorubicin by drug-resistant cell lines and their parent cell lines. Both type of cells were treated with 5 μM DOX for 12 h. Subsequently, observations were made using a confocal microscope. The red fluorescence received by the mCherry channel was emitted by the excitation of doxorubicin by a 587 nm laser.

Furthermore, doxorubicin is capable of autofluorescence after excitation with excitation light and is a commonly used model drug. Therefore, we observed the uptake of doxorubicin by CMT-7364/R and its parental cell lines after 12 h. The results are shown in Figure 5. Among them, the red fluorescence received by the mCherry channel is doxorubicin. It can be seen that doxorubicin is hardly visible in the Merge images taken after 12 h doxorubicin treatment for CMT-7364/R. And combined with the localization effect of DAPI, it can be clearly seen that the doxorubicin has been taken up into CMT-7364/S. This indicates that the uptake of doxorubicin by CMT-7364/S is stronger. The above results, in many ways, verified the resistance of CMT-7364/R to doxorubicin.

**Figure 6:**
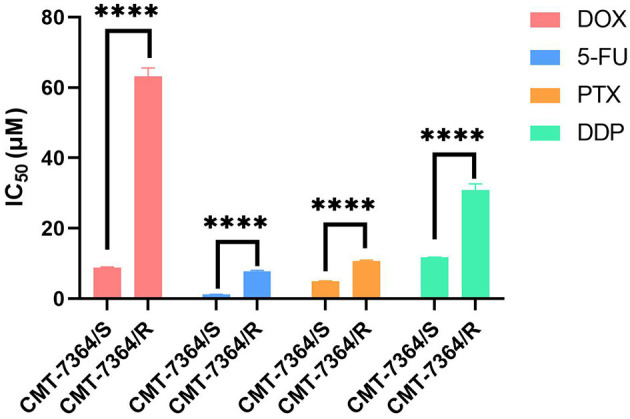
The IC_50_ of various clinically commonly used chemotherapy drugs for drug-resistant cell lines and their parent cell lines. DOX, doxorubicin; 5-Fu, 5-fluorouracil; PTX, Paclitaxel; DDP, Cisplatin. Data were presented as the mean ± SD of three independent experiments. ****p < 0.0001.

To verify whether CMT-7364/R is multi-drug resistant, we also examined the IC_50_ of several other drugs commonly used clinically for chemotherapy of canine mammary tumors on parental and resistant cell lines, including 5-fluorouracil (5-FU), paclitaxel (PTX), and cisplatin (DDP), and the results of their IC_50_ are shown in Figure 6. The resistance index of CMT-7364/R to the above three drugs (RI) were 6.779, 2.200, and 2.634, respectively, indicating that CMT-7364/R had multi-drug resistance to the above drugs.

### 3.3. Drug-resistant cell line has higher expression level of Breast Cancer Resistance Protein

**Figure 7:**
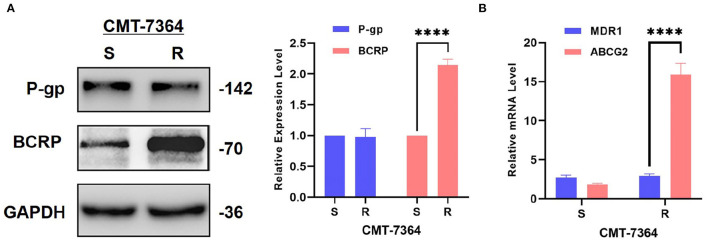
Expression level of P-gp and BCRP in drug-resistant cell lines and their parent cell lines. **(A)** Western Blot and semi-quantitative analysis. **(B)** RT-qPCR. The results showed that the transcript and protein expression levels of BCRP were significantly higher in CMT-7364/R compared to CMT-7364/S. GAPDH was used as an internal control. Data were presented as the mean ± SD of three independent experiments. ****p < 0.0001.

**Figure 8:**
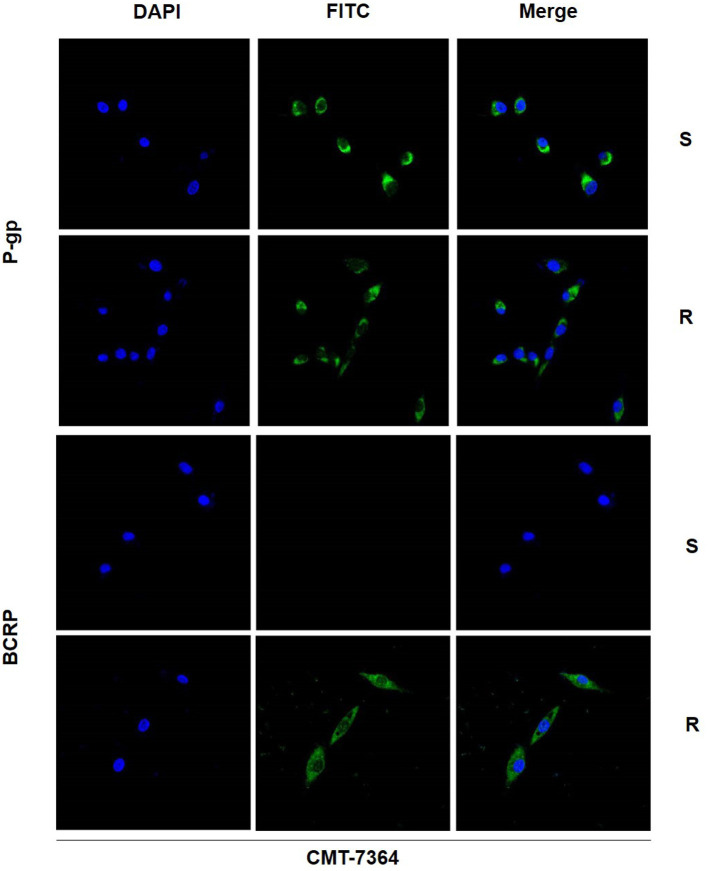
Immunofluorescence images of P-gp and BCRP in drug-resistant cell lines and parental cell lines. The green fluorescence shown by FITC is the target protein, and the blue fluorescence shown by DAPI is used for localization to the nucleus. S, CMT-7364/S; R, CMT-7364/R. The protein expression of BCRP was significantly elevated in drug-resistant cell lines compared to parental cell lines.

There are numerous mechanisms by which tumor cells develop multi-drug resistance, and one of the most common mechanisms is the presence of over-expression of ABC transporter pumps. To investigate the mechanisms by which CMT-7364/R develops multi-drug resistance, we examined two common ABC transporter proteins, including P-gp and BCRP. No significant difference was seen in the expression level of P-gp in CMT-7364/R compared to its parental cell line, while the expression level of BCRP was significantly elevated (Figure 7). By semi-quantitative analysis, the differences in their relative expression levels were seen to be statistically significant (****p < 0.0001). In addition, we examined the differences in transcript levels of these two genes by RT-qPCR, and the results are shown in the Figure 7 as well, indicating that they are also significantly different in transcript levels (****p < 0.0001). To more visually verify the expression of P-gp with BCRP, we performed immunofluorescence assay and observed the expression of P-gp and BCRP among the cells using confocal microscopy, as shown in Figure 8. P-gp was expressed in both CMT-7364/S and CMT7364/R, while BCRP was only significantly expressed in CMT-7364/R. Combining the above results of the two ABC transporter pumps at the transcriptional as well as protein levels, we can conclude that there is no significant difference in the expression of P-gp in CMT-7364/S versus CMT-7364/R, while BCRP is highly expressed in CMT-7364/R.

### 3.4. Drug-resistant cell line has an increased ability of invasion and metastasis compared to their parental cell lines

**Figure 9:**
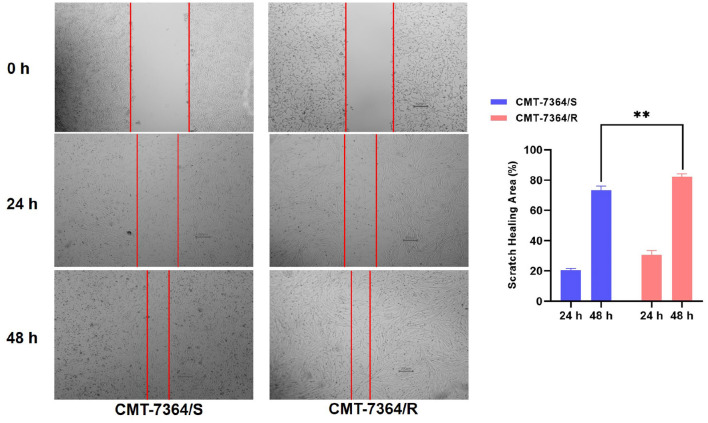
Migration of drug-resistant cell lines and their parental cell lines and percentage of scratch healing area. After 24 h and 48 h, the healing area of the scratches were measured, and after 48 h, the percentage of healed scratches in the CMT-7364/R group was 82.30 ± 1.054, while that in the CMT-7364/S group was 73.30 ± 1.600. Data were presented as the mean ± SD of three independent experiments. **p < 0.01.

**Figure 10:**
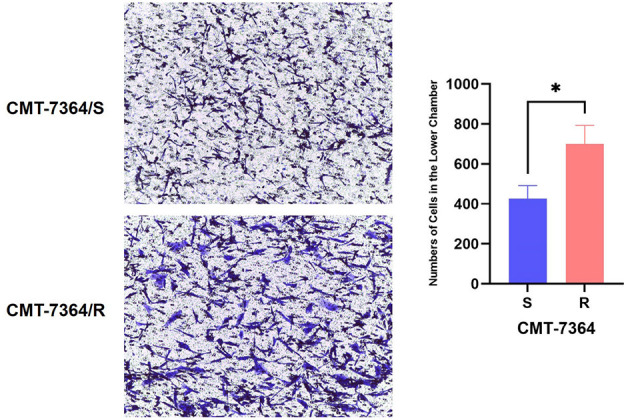
Number of cells in which drug-resistant cell lines and their parent cell lines cross the stromal membrane. The pore size of the Transwell chamber used in this experiment is 8.0 μm. After 24 h, the number of cells invading the lower chamber was 700 ± 54 and 426 ± 38 cells in the CMT-7364/R and parental cell line groups, respectively. Data were presented as the mean ± SD of three independent experiments. *p < 0.05.

Given the mesenchymal changes in the morphology of the cells, we hypothesized that the migration and invasive ability of the cells might be altered. Firstly, the results of the cell scratch healing assay showed that the difference in the area of scratch healing due to cell migration was statistically significant in CMT-7364/R compared to the parental cell line aft. After 48 h, the percentage of scratch healing area in the CMT-7364/R group was 82.30 ± 1.054 compared with 73.30 ± 1.600 in the CMT-7364/S group (**p < 0.01). This result indicated that the migration ability of CMT-7364/R was enhanced compared to CMT-7364/S (Figure 9). Secondly, to compare the differences in invasive capacity between CMT-7364/R and its parental cell lines, we performed Transwell invasion assays. The invasive ability of tumor cells was assessed by counting the number of cells that crossed the matrix gel and the polycarbonate membrane to reach the outer surface of the membrane (Figure 10). After 24 h, the number of cells that invaded into the lower chamber was 700 ± 54 as well as 426 ± 38 cells in CMT-7364/R and its parental cell line groups, respectively (*p < 0.05). This result indicated that the invasion ability of CMT-7364/R was enhanced compared to CMT-7364/S.

**Figure 11:**
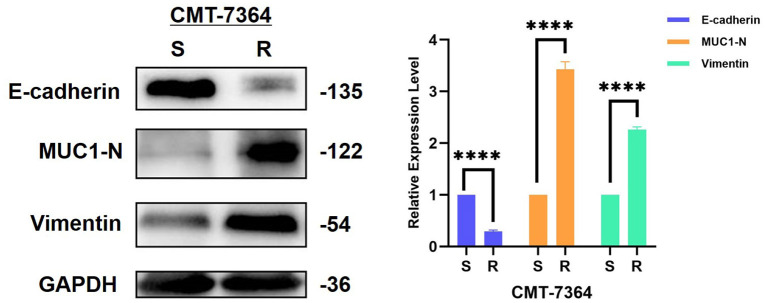
Expression of EMT-related proteins in drug-resistant cell lines and their parent cell lines. The results showed that the expression level of E-cadherin was reduced in the resistant cell line compared to the parental cell line, while the expression levels of MUC1-N and vimentin were increased, consistent with the characteristics of EMT. GAPDH was used as an internal control. Data were presented as the mean ± SD of three independent experiments. ****p < 0.0001, “ns” stands for not significant.

From the above results, it is clear that the invasion and metastatic ability of CMT-7364/R is significantly enhanced compared to its parental cell line, and combined with its morphological changes, we hypothesized that CMT-7364/R may have undergone epithelial-mesenchymal transition (EMT) during the process of high-dose drug pulse development. For this purpose, we examined the expression of the relevant proteins, and the results are shown in Figure 11. The expression level of E-cadherin was significantly reduced in the drug-resistant cell line, while the expression level of vimentin was significantly increased (****p < 0.0001), which are consistent with the expression profile of the cell line in which the EMT process occurred. Mucin 1 (MUC1) is a transmembrane protein of the mucin family, consisting of an extracellular N-terminal and an intracellular C-terminal, which can form heterodimers; the longer N-terminal peptide acts as a physical barrier and is significantly correlated with cell adhesion, migration, and invasion. As we mentioned above the difference in adhesion ability between the two cell lines, we examined the expression level of MUC1-N. As a result, the expression level of MUC1-N was significantly elevated in CMT-7364/R (Figure 11).

### 3.5. Transcriptomes analysis identifies different gene expression between the parental and drug-resistant cell lines

**Figure 12:**
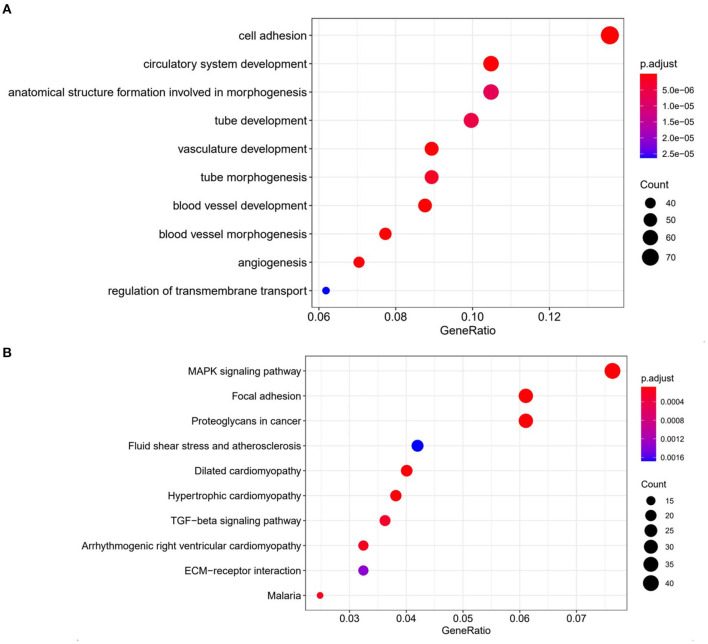
Comparison of transcriptome expression profiles of drug-resistant cell lines and parental cell lines. **(A)** GO analysis shows that biological functions related to cell adhesion capacity are elevated in drug-resistant cell lines compared to parental cell lines. **(B)** KEGG analysis showed that the MAPK pathway was activated in drug-resistant cells compared to parental cells.

To investigate the potential mechanism of the development of drug-resistance in CMT-7364/R, we analyzed transcriptome expression between drug-resistant cells and parental cells by RNA-sequencing. GO analysis showed a significant improvement in the cell adhesion capacity of CMT-7364/R, which is consistent with the phenomenon we observed in cell culture. According to the results of KEGG analysis, the MAPK pathway of CMT-7364/R is activated (Figure 12).

### 3.6. Tumorigenic analysis

**Figure 13:**
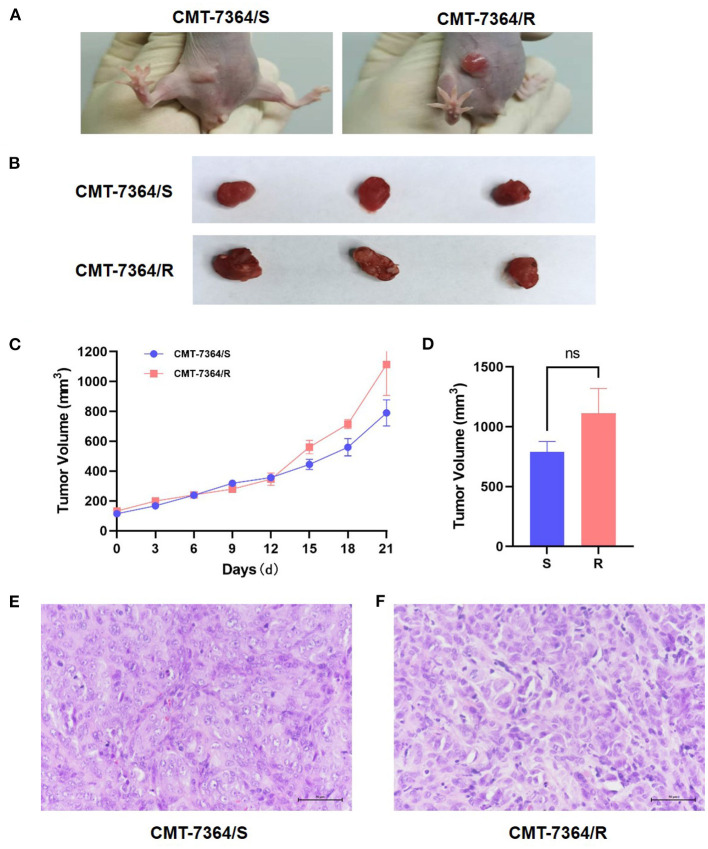
Mouse xenograft models. **(A)** The appearance of masses on the 10th day after inoculation of tumor cells. Notice that the mass formed by CMT-7364/S shows limited growth, clear boundaries and intact epidermis while the tumor formed by CMT-7364/R has a variable appearance and often ulcers. **(B)** The appearance of masses at the end of the study. **(C)** Volume growth curve of masses formed by drug-resistant cell lines and their parent cell lines. **(D)** The final volume of the masses. **(E)** The histopathological image of CMT-7364/S with scale bar of 50 μm. **(F)** The histopathological image of CMT-7364/R with scale bar of 50 μm. Data were presented as the mean ± SD of three independent experiments. “ns” stands for not significant.

In this study, xenograft models of both drug-resistant cell lines and parental cell lines were constructed. After inoculating tumor cells for 5 days, gradually enlarging solid mass with a clear outline in the mammary area on the left side of the groin can be seen in mice injected with either the drug-resistant cell line or the parental cell line. However, the masses that grow in mice inoculated with CMT-7364/R are larger in size and often ulcerate. Within 21 days, the volume of the mass was recorded every 3 days. After 21 days, the mice were sacrificed, and the final volume of the mass is measured (Figure 13). Masses caused by CMT-7364/R grew faster than masses caused by their parental cell lines, but the volume of the masses caused by both cell lines have no significant difference at the end of the study.The histopathological examination by H&E staining showed that the swelling caused by CMT-7364/R was also ductal papillary epithelial carcinoma, which was consistent with its parental cell line and could serve as a verification of their homology. The epithelial cells of the tumor were multilayered and had malignant features with moderate duct formation, remarkable nuclear heterogeneity, and mitotic phase.

## 4. Discussion

CMTs are the most common tumors in female dogs, and the limited treatment options are a major cause for the poor prognosis of malignant CMTs, while adjuvant chemotherapy remains the most commonly used systemic therapy for CMTs. However, CMTs often develope drug resistance to cytotoxic agents. There is an urgent need to elucidate the molecular mechanism of chemoresistance in CMTs. However, the mechanisms of drug resistance in CMTs are complex and result from multiple factors and signaling pathway interactions. The adenosine triphosphate-binding cassette (ABC) transporter protein, which uses ATP to pump chemotherapeutic drugs out of the cell, is a common mechanism of resistance, and overexpression of P-gp and BCRP is most frequently seen in canine breast cancers that develop multi-drug resistance. Completely unraveling this network remains a major challenge and is essential for identifying new therapeutic targets. However, in order to understand the mechanisms involved, stable cell models are urgently needed. In human medicine, *in vitro* drug-resistant cell models have been established for several kinds of tumors by means of *in vitro* construction, including breast cancer, liver cancer, colon cancer, and melanoma, among others (13). In contrast, the field of veterinary research lacks corresponding but necessary research materials.

Thus, this study developed a canine mammary tumor drug-resistant cell line by high-dose drug pulse method. During *in vitro* development, CMT-7364 initially showed massive cell population death after drug stimulation, and large areas of cell de-adhesion were visible microscopically, floating in the culture medium. Very few cell populations remained adherent and proliferative after elution. After several months of induction by continuous high-dose drug pulse method, CMT-7364 gradually developed resistance to the drug and could still adhere to the bottom of the dish after receiving the same dose of drug. Under the optical microscope, the cells gradually developed from a shuttle shape with clear borders initially to a morphology with unclear borders and a strong anisotropic phenotype. By the CCK-8 method, we confirmed that the cells were multi-drug resistant and named the obtained resistant cell line as CMT-7364/R and the parental cell line as CMT-7364/S.

We first compared the proliferative capacity of the two cell lines, and by comparing the PDT of both, we found that the proliferative capacity of CMT-7364/R was significantly reduced. In addition, doxorubicin has a strong antitumor effect as a cycle non-specific anticancer chemotherapeutic agent that causes G2/M phase block of the cell cycle (14). And by comparing the cell cycle distribution of both cells after treatment with 5 μM and 10 μM DOX for 24h, we found that CMT-7364/R not only had stronger resistance to the G2/M phase blocking effect of DOX, but also the percentage of cells located in G0/G1 phase was higher compared to CMT-7364/S. In combination with the above results, we hypothesize that since cytotoxic drugs are usually most effective on rapidly dividing cells (15), this near “dormant” behavior may instead provide them with a natural defense against cytotoxic drugs, allowing these slower proliferating cells sufficient time to repair the DNA damage caused by DOX. This is one of the reasons for the resistance of CMT-7364/R to DOX (16).

Secondly, since overexpression of the ABC transporter pump is one of the common mechanisms mediating multi-drug resistance in tumors, we examined the expression of P-gp, which plays a major role in breast cancer together with BCRP, the most well-characterized drug transporter in the ABC protein superfamily, encoded by the ABCB1 (formerly known as MDR1). Among human medical oncology cases, P-gp is probably best known for its role in mediating multi-drug resistance to chemotherapeutic drugs. In recent years, tumor resistance has begun to gain attention in the veterinary field due to a aging trend in the average age of companion animals and an increase in tumor incidence (17, 18). Currently, there are commercial rapid assays available to detect P-gp expression for prognosis of adjuvant chemotherapy and to avoid toxicity of P-gp substrate drugs in dogs with MDR1 mutations, especially in Collies. However, the same attention for BCRP is insuffient. In this study we found that there was no significant difference in P-gp expression between CMT-7364/R and CMT-7364/S, whereas BCRP was significantly elevated in CMT-7364/R both at the transcriptional and protein expression levels and was another important cause of multi-drug resistance in CMT-7364/R. This finding suggests the importance of BCRP in multi-drug resistance in canine mammary tumors. The simultaneous inclusion of BCRP expression with P-gp is relevant for the prognostic of adjuvant chemotherapy in small animal clinical canine mammary tumors.

Finally, we examined the migration and invasion ability of both cell types by scratch healing assay and Transwell invasion assay. From these results, it is clear that the invasion and metastatic ability of CMT-7364/R was significantly enhanced compared with its parental cell line, and combined with its morphological changes, we speculate that CMT-7364/R may have undergone epithelial-mesenchymal transition (EMT) during induction by high-dose drug pulse *in vitro*. EMT is an important process in normal embryonic development and is the most common cause of tumor invasion and metastasis initiation causes (19). Typical EMT cells increase motility and migratory capacity by altering the extracellular matrix, as evidenced by lysis of epithelial intercellular junctions, impaired basement membrane integrity, altered cell polarity and cytoskeletal rearrangements, decreased expression of E-cadherin, a shift from predominantly keratin to vimentin expression, loss of the typical polygonal, cobblestone-like epithelial cell appearance, a spindle-shaped fibroblast-like appearance and expression of mesenchymal cell markers and matrix metalloproteinase activity (20), for which we examined the expression of the relevant proteins. The expression level of E-cadherin were significantly reduced in the drug-resistant cell lines, while the expression level of vimentin significantly increased. We were not able to detect N-cadherin signals in CMT-7364/R or CMT-7364/S, probably due to the fact that N-cadherin is mostly expressed in cells such as cardiac muscle and smooth muscle. A recent study examined the role of E-cadherin in metastasis using mouse and human models of tubular and basal infiltrative ductal carcinoma (21). The results showed that E-cadherin promotes metastasis in different models of invasive ductal carcinoma. Although E-cadherin deficiency increased the invasive ability of tumor cells, it also reduced tumor cell proliferation, the number of circulating tumor cells, tumor cell colonization of distant organs, and the growth of metastatic site. This is consistent with the phenotype we observed in CMT-7364/R. In addition, mucin-1 has a longer extracellular N-terminal end, which can act as a physical barrier and correlates with cell migration and invasion (22). It was also significantly highly expressed in CMT-7364/R. Based on the above results, we speculate that the CMT-7364/R cell line may be at the stage of epithelial-mesenchymal transition and the high expression of MUC1-N may act as a physical barrier, which is one of the reasons for its drug resistance.

Overall, we constructed a multi-drug resistant cell line, CMT-7364/R, indicating that it may serve as a useful model for future studies on tumor drug resistance. In the field of human medicine, tumor-resistant cell lines have been widely used to investigate the mechanisms of drug resistance development and the reversal of drug resistance (6, 23). While the use of multi-drug resistant cell lines in veterinary medicine is still a relatively new area of research, there are several potential applications for our experimental tool. For example, the CMT-7364/R cell line could be used to investigate drug resistance mechanisms specific to canine mammary tumors. This could provide insights into potential therapeutic targets for this disease and help to develop more effective and personalized treatment options for dogs with multi-drug resistant mammary tumors. Additionally, our experimental tool could be used to evaluate the efficacy of new drug candidates for canine mammary tumors. By testing new drugs or drug combinations on the CMT-7364/R cell line, researchers could identify promising treatment options that could then be further evaluated in clinical trials. Finally, the use of *in vitro* canine breast cancer cell lines and naturally occurring canine mammary tumors has proven to be a valuable tool for advancing our understanding of animal cancer and can also serve as excellent translational models for human breast cancer (24). Currently, a study have constructed an *in vitro* canine mammary tumor cell line that has acquired radiotherapy resistance, which has been used to compare with human breast cancer cell lines to investigate the mechanism of acquired radiotherapy resistance (18). Thus, through comparative oncology studies, the drug-resistant cell lines we have constructed may help to improve the understanding of drug resistance of human breast cancer (25).

## 5. Conclusion

In our study, we constructed a canine mammary tumor cell line CMT-7364/R by high-dose drug shock *in vitro*. Compared to its parental cell line, CMT-7364-R is multi-drug resistant, which may be associated with lower proliferation rate, the overexpression of BCRP and enhanced migration and invasion ability. The use of this drug-resistant cell line may contribute to future investigations of the development of drug resistance in canine mammary tumors.

## Data availability statement

The datasets presented in this study can be found in online repositories. The names of the repository/repositories and accession number(s) can be found in the article/supplementary material.

## Ethics statement

The animal study was reviewed and approved by the Animal Ethics Committee of China Agricultural University.

## Author contributions

CZ and DZ designed the whole study. CZ performed the experimental work and data analysis and wrote the manuscript. ZL, XL, and PS took part in some of the experiments. ZL and DZ revised the manuscript. All authors read and approved the manuscript.

## References

[1] Campbell MM. Pachyderms, primates, plants and population. Reproduct Fertility Dev. (2001) 13:697. 10.1071/RD01069 11999323

[2] Goldschmidt MH Peña L Zappulli V. Tumors of the mammary gland. In: Tumors in Domestic Animals. New Jersey: John Wiley & Sons, Inc. (2016). p. 723–65.

[3] Salas Y Márquez A Diaz D Romero L. Epidemiological study of mammary tumors in female dogs diagnosed during the period 2002-2012: a growing animal health problem. PLoS ONE. (2015) 10:e0127381. 10.1371/journal.pone.0127381 25992997 PMC4436381

[4] Sorenmo KU Worley DR Zappulli V. Tumors of the mammary gland. In: Withrow and MacEwen's Small Animal Clinical Oncology. Amsterdam (2019). p. 604–25.

[5] Longley D Johnston P. Molecular mechanisms of drug resistance. J Pathol. (2005) 205:275–92. 10.1002/path.1706 15641020

[6] Vasan N Baselga J Hyman DM. A view on drug resistance in cancer. Nature. (2019) 575:299–309. 10.1038/s41586-019-1730-1 31723286 PMC8008476

[7] Robey RB Hay N. Is Akt the “Warburg kinase”?–Akt-energy metabolism interactions and oncogenesis. Semin Cancer Biol. (2009) 19:25–31. 10.1016/j.semcancer.2008.11.010 19130886 PMC2814453

[8] Sharom FJ. ABC multidrug transporters: structure, function and role in chemoresistance. Pharmacogenomics. (2008) 9:105–27. 10.2217/14622416.9.1.105 18154452

[9] McDermott M Eustace AJ Busschots S Breen L Crown J Clynes M. *In vitro* development of chemotherapy and targeted therapy drug-resistant cancer cell lines: a practical guide with case studies. Front Oncol. (2014) 4:40. 10.3389/fonc.2014.00040 24639951 PMC3944788

[10] Liang XJ Shen DW Garfield S Gottesman MM. Mislocalization of membrane proteins associated with multidrug resistance in cisplatin-resistant cancer cell lines. Cancer Res. (2003) 63:5909. 14522917

[11] Rahmanian M Seyfoori A Ghasemi M Shamsi M Kolahchi AR Modarres HP. In-vitro tumor microenvironment models containing physical and biological barriers for modelling multidrug resistance mechanisms and multidrug delivery strategies. J Control Release. (2021) 334:164–77. 10.1016/j.jconrel.2021.04.024 33895200

[12] Zhang H Pei S Zhou B Wang H Du H Zhang D. Establishment and characterization of a new triple-negative canine mammary cancer cell line. Tissue Cell. (2018) 54:10–19. 10.1016/j.tice.2018.07.003 30309498

[13] Hamed AR Abdel-Azim NS Shams KA Hammouda FM. Targeting multidrug resistance in cancer by natural chemosensitizers. Bull Natl Res Centre. (2019) 43:8. 10.1186/s42269-019-0043-8

[14] Kim HS Lee YS Kim DK. Doxorubicin exerts cytotoxic effects through cell cycle arrest and fas-mediated cell death. Pharmacology. (2009) 84:300–9. 10.1159/000245937 19829019

[15] Shibata M Hoque MO. Targeting cancer stem cells: a strategy for effective eradication of cancer. Cancers. (2019) 11:732. 10.3390/cancers11050732 31137841 PMC6562442

[16] Rehman SK Haynes J Collignon E Brown KR Wang Y Nixon A. Colorectal cancer cells enter a diapause-like DTP state to survive chemotherapy. Cell. (2021) 184:226–42.e21. 10.1016/j.cell.2020.11.018 33417860 PMC8437243

[17] Valdivia G Alonso-Diez N Pérez-Alenza D Pea L. From conventional to precision therapy in canine mammary cancer: a comprehensive review. Front Vet Sci. (2021) 8:623800. 10.3389/fvets.2021.623800 33681329 PMC7925635

[18] Gray M Turnbull AK Meehan J Martínez-Pérez C Argyle DJ. Comparative analysis of the development of acquired radioresistance in canine and human mammary cancer cell lines. Front Vet Sci. (2020) 7:439. 10.3389/fvets.2020.00439 32851022 PMC7396503

[19] Raposo-Ferreira TMM Brisson BK Durham AC Laufer-Amorim R Kristiansen V Puré E. Characteristics of the epithelial-mesenchymal transition in primary and paired metastatic canine mammary carcinomas. Vet Pathol. (2018) 55:622–33. 10.1177/0300985818776054 29788797 PMC7993492

[20] Klymkowsky MW Savagner P. Epithelial-mesenchymal transition. Am J Pathol. (2009) 174:1588–93. 10.2353/ajpath.2009.080545 19342369 PMC2671246

[21] Padmanaban V Krol I Suhail Y Szczerba BM Aceto N Bader JS. E-cadherin is required for metastasis in multiple models of breast cancer. Nature. (2019) 573:439–44. 10.1038/s41586-019-1526-3 31485072 PMC7365572

[22] Vafaei R Samadi M Hosseinzadeh A Barzaman K Esmailinejad M Khaki Z. Comparison of mucin-1 in human breast cancer and canine mammary gland tumor: a review study. Cancer Cell Int. (2022) 22:14. 10.1186/s12935-021-02398-6 35000604 PMC8744232

[23] Jiang L Wang P Sun YJ Wu YJ. Ivermectin reverses the drug resistance in cancer cells through EGFR/ERK/Akt/NF-κB pathway. J Exp Clin Cancer Res. (2019) 38:1–18. 10.1186/s13046-019-1251-7 31215501 PMC6580523

[24] Gray M Meehan J Martínez-Pérez C Kay C Turnbull AK Morrison LR. Naturally-occurring canine mammary tumors as a translational model for human breast cancer. Front Oncol. (2020) 10:617. 10.3389/fonc.2020.00617 32411603 PMC7198768

[25] LeBlanc AK Mazcko CN. Improving human cancer therapy through the evaluation of pet dogs. Nat Rev Cancer. (2020) 20:727–42. 10.1038/s41568-020-0297-3 32934365

